# Circulating Gene Expression Assay as a Diagnostic and Prognostic Biomarker for Pancreatic Neuroendocrine Tumors in MEN1

**DOI:** 10.1210/clinem/dgaf374

**Published:** 2025-06-24

**Authors:** Eline N M van Vliembergen, Mark J C van Treijen, Rachel S van Leeuwaarde, Carolina R C Pieterman, Joanne M de Laat, Margot E T Tesselaar, Gerlof D Valk

**Affiliations:** Department of Endocrine Oncology, University Medical Center Utrecht, 3584 CX Utrecht, The Netherlands; Department of Endocrinology, Radboud University Medical Center, 6525 GA Nijmegen, The Netherlands; Department of Endocrine Oncology, University Medical Center Utrecht, 3584 CX Utrecht, The Netherlands; Center for Neuroendocrine Tumours, ENETS Center of Excellence, Netherlands Cancer Institute, University Medical Center Utrecht, 3584 CX Utrecht, The Netherlands; Department of Endocrine Oncology, University Medical Center Utrecht, 3584 CX Utrecht, The Netherlands; Center for Neuroendocrine Tumours, ENETS Center of Excellence, Netherlands Cancer Institute, University Medical Center Utrecht, 3584 CX Utrecht, The Netherlands; Department of Endocrine Oncology, University Medical Center Utrecht, 3584 CX Utrecht, The Netherlands; Center for Neuroendocrine Tumours, ENETS Center of Excellence, Netherlands Cancer Institute, University Medical Center Utrecht, 3584 CX Utrecht, The Netherlands; Department of Endocrine Oncology, University Medical Center Utrecht, 3584 CX Utrecht, The Netherlands; Department of Endocrinology, Radboud University Medical Center, 6525 GA Nijmegen, The Netherlands; Center for Neuroendocrine Tumours, ENETS Center of Excellence, Netherlands Cancer Institute, University Medical Center Utrecht, 3584 CX Utrecht, The Netherlands; Department of Medical Oncology, Netherlands Cancer Institute, 1066 CX Amsterdam, The Netherlands; Department of Endocrine Oncology, University Medical Center Utrecht, 3584 CX Utrecht, The Netherlands; Center for Neuroendocrine Tumours, ENETS Center of Excellence, Netherlands Cancer Institute, University Medical Center Utrecht, 3584 CX Utrecht, The Netherlands

**Keywords:** multiple endocrine neoplasia type 1, pancreatic neuroendocrine tumors, biomarker, gene assay

## Abstract

**Objective:**

There is an unmet need for biomarkers in multiple endocrine neoplasia type 1 (MEN1)-related pancreatic neuroendocrine tumors (PanNETs) that allow prediction of clinical behaviour and metastatic potential. This study aims to investigate the potential of a circulating NET mRNA gene expression assay as a diagnostic and prognostic biomarker.

**Design:**

Single-center, prospective, cohort study.

**Methods:**

MEN1 patients were enrolled between July 2016 and June 2017. Blood samples were collected in PAXgene tubes and the original NETest assay recalibrated for MEN1 PanNETs. The mNET assay was performed at baseline. Patients were followed for 39 months (range 12-48 months). Diagnostic and predictive values were assessed using the area under the receiver operating characteristic curve (AUC).

**Results:**

Of 110 eligible patients, 60% were diagnosed with PanNETs at baseline and 9% developed PanNETs during follow-up. At baseline, the mNET assay differentiated between patients without manifestations and patients with only PanNETs (*P* = .04). The AUC for predicting PanNET development was 0.65 (*P* = .15). In separate analyses, the assay did not predict PanNET growth (AUC = 0.49), number of PanNETs (AUC = 0.54), new metastatic disease (AUC = 0.39), or metastasis progression (AUC = 0.46).

**Conclusion:**

In line with previous studies in non-MEN1 associated NET, in MEN1, a circulating NET gene expression assay identified patients without any manifestations from PanNETs. The mNET assay did not predict PanNET development, progression, or metastasis. The underlying genetic condition, epigenetic modifications, microadenomas, and coexistence of multiple manifestations may impact circulating RNA profiles. Further research should explore alternative biomarkers or develop clinico-molecular algorithms for personalized management strategies in MEN1.

Multiple endocrine neoplasia type 1 (MEN1) is a hereditary endocrine tumor syndrome caused by a germline mutation on chromosome 11q13 in the MEN1 tumor suppressor gene, encoding menin (1). The syndrome is characterized by the multiple occurrence of endocrine neoplasms of the parathyroid glands, pancreas, and anterior pituitary gland (2). Beyond these 3 major manifestations, individuals with MEN1 also develop neuroendocrine tumors of the lung, thymus, gastrointestinal tract, and adrenal cortex (2). The lifetime risk of developing pancreatic neuroendocrine tumors (PanNETs) exceeds 80%, and metastatic disease originating from these tumors is the primary cause of a reduced life expectancy by 15 years (3-6). In MEN1, PanNETs are often multiple, and the majority of PanNETs are nonfunctioning (7).

Pancreatic imaging is the cornerstone of surveillance programs for nonfunctioning PanNETs (2, 8). The aim of surveillance is early detection and precise monitoring of PanNET growth to enable treatment before metastasis occurs and to reduce PanNET-associated morbidity and mortality (2). Due to the multiplicity of PanNETs, variation in growth potential, and their unpredictable malignant behavior, clinical decision-making is challenging with regard to timing and the extent of interventions (often surgery). Currently, tumor size appears to best correlate with metastatic potential. There is consensus to use a wait-and-see policy for PanNETs up to a size of 2 cm, with surgical resection recommended once the size exceeds this threshold or in cases of rapid growth (8). However, even though the majority of small tumors appear indolent for years, PanNETs can metastasize before reaching 2 cm (4, 9, 10). Therefore, an intensive and lifelong imaging-based surveillance program is advised in all MEN1 patients, starting from childhood.

Hence, there is an unmet clinical need for prognostic biomarkers to identify MEN1 patients who are prone to an aggressive disease course, thereby enabling personalized PanNET treatments. These biomarkers should enable timely intervention for patients who have PanNETs with aggressive behavior while minimizing unnecessary and expensive screening scans for patients with indolent tumors, as is suggested by expert panels and in multiple publications (11-14). Chromogranin A, pancreatic polypeptide, and glucagon have been assessed as biomarkers, but their individual and combined accuracy for assessing PanNET in MEN1 is low (15, 16). An emerging biomarker under consideration is the NETest, an algorithmic assay quantifying circulating genetic information of 51 neuroendocrine neoplasms-related genes in blood by quantitative PCR (17). In multiple studies, the NETest proved to be very promising (18). The applicability of the NETest for MEN1 is unknown. Given the unpredictable disease course and the substantial burden associated with frequent screening scans, a circulating gene expression assay could offer a much-awaited solution for this population. Consequently, it is important to assess whether it could be used for predicting the behavior of PanNET in MEN1. Therefore, this study aims to assess the assay as a diagnostic and prognostic marker of the clinical behavior of PanNETs in a large cohort of MEN1 patients.

## Methods

MEN1 patients treated at the University Medical Center Utrecht and the Netherlands Cancer Institute (Amsterdam) were prospectively enrolled between July 2016 and June 2017. Written informed consent was obtained from all participants involved, both in the training set and the validation set, for collecting data and blood samples. The research was approved by the local ethics committee and conducted in accordance with the Declaration of Helsinki. Patients were followed up for a duration ranging from 12 to 48 months, with a median follow-up period of 39 months. Individuals lost to follow-up within 12 months after blood sampling for the mNET assay were excluded from the analysis.

Peripheral blood samples were obtained at the time of enrollment using PAXgene tubes and stored at −80°C within 2 hours of collection. The collected tubes were deidentified and anonymously coded before being dispatched to Wren Laboratories (Branford, CT, USA), where the mNET assay was conducted.

### Radiological Evaluation of PanNETs

The diagnosis of a PanNET was established through a thorough assessment of anatomical and functional imaging by specialized radiologists experienced in pancreatic imaging for MEN1 patients. The lesions had to be consistently identified on consecutive imaging scans. Follow-up imaging was undertaken as dictated by the patients' clinicians, according to the practice guidelines (2, 8).

Magnetic resonance imaging (MRI) was the preferred imaging modality for the assessment of PanNET progression. In cases where MRI was contraindicated, a computed tomography (CT) scan was performed.

A PanNET was considered stable if no radiological progression was noted or when the calculated growth was less than 1.0 mm per year. Conversely, progressive disease was identified when there was growth of at least 1.0 mm per year over 3 consecutive years or when new PanNETs were detected on at least 2 consecutive scans. The Response Evaluation Criteria In Solid Tumors were not utilized since they are based on tumors with rapid progression and insufficiently define progression in PanNETs, given the more indolent nature of these tumors (9, 19). Similarly, the Choi criteria were found unsuitable (20). The definition of progression in PanNETs remains a topic of ongoing debate, and no universally accepted criteria currently exist. The threshold of 1.0 mm per year was selected based on a previous study, which found that progressive tumors grew at an average rate of 0.39 mm per quarter (ranging from 0.28 to 0.49 mm) (9).

If a patient underwent pancreatic surgery or peptide receptor radionuclide therapy (PRRT) during the follow-up period, the PanNET was deemed progressive if there was evidence of growth before the intervention. Patients who underwent imaging only once before these interventions were excluded because of the unknown growth status prior to intervention.

Metastatic disease was established either through histopathological examination or, when not histologically confirmed, based on metastatic disease observed on MRI or CT with a nonphysiologically increased uptake at ^68^Ga-DOTA-TOC PET/CT, according to assessment by specialized radiologists and recorded on at least 2 consecutive imaging studies. The progression of metastatic disease was defined as either the emergence of new metastases, as confirmed on at least 2 consecutive scans, or the annual growth of preexisting metastatic lesions by at least 1 mm.

### The NET Gene Expression Assay

The original NETest is a blood-based genetic biomarker assay that quantifies 51 circulating neuroendocrine tumor transcripts, correlating with the tumor tissue expression levels (21, 22). The test comprises a 2-step protocol: RNA isolation/cDNA production and a quantitative PCR. The resulting score is generated through a set of machine learning algorithms. The NETest methodology has been described in detail in previous publications (21, 22).

The original algorithms and scoring were developed on gene expression measured in samples collected using EDTA tubes and then updated to an RNA stabilization tube collection protocol. PAXgene tubes are also frequently used to stabilize mRNA and may provide an alternative collection approach. PAXgene tubes are superior to EDTA tubes in stabilization of circulating RNA, resulting in higher amounts of circulating neuroendocrine tumor transcripts with altered ratios. Consequently, the algorithm developed for EDTA tubes was no longer applicable. Given the potential gene expression differences in blood collected in PAXgene tubes compared to EDTA tubes, the NETest assay was recalibrated at Wren Laboratories in a 2-step process before being used for the study.

In step 1, blood in PAXgene tubes from healthy controls was collected (n = 23) and compared with PAXgene expression levels of 5 sporadic gastroenteropancreatic NET patients. This was to identify a baseline gene expression for controls compared to NETs (no MEN1 germline mutation) in PAXgene collected samples.

In step 2, the algorithms were recalibrated to differentiate PanNETs from non-PanNETs in patients with a germline MEN1 mutation. For this, a training set of 15 MEN1 patients with PanNETs and 15 MEN1 patients with no manifestations was evaluated using the original 4 machine learning algorithms (23). These patients were excluded from the final analysis.

This PAXgene-collected, MEN1 refocused NETest is scored 0% to 100%, has a cut-off of normal of 20%, and is labeled as the “mNET assay.” The 20% cut-off was determined using the Youden index, which identifies the optimal threshold based on the area under the curve (AUC) of the receiver operating characteristics curve.

### Statistics

Clinical characteristics are presented as mean values with SDs or medians with ranges, depending on the distribution of the data. Continuous data were compared using the independent *t*-test, the Mann–Whitney U-test, or the Kruskal–Wallis test, depending on the data distribution. Post hoc analyses were performed using Dunn's procedure with a Bonferroni correction. Categorical data were analyzed using Fisher's exact test or the chi-square test, depending on the number of categories involved. The accuracy of the mNET assay was evaluated through the AUC of the receiver operating characteristics, the sensitivity, and the specificity. To assess the accuracy of the mNET assay in predicting tumor growth, only patients with PanNETs at baseline were included in the analysis. For the other analyses, the entire study population was included. Statistical significance was defined at a *P*-value <.05. Statistical analyses were performed using IBM SPSS version 29.

## Results

**Figure 1:**
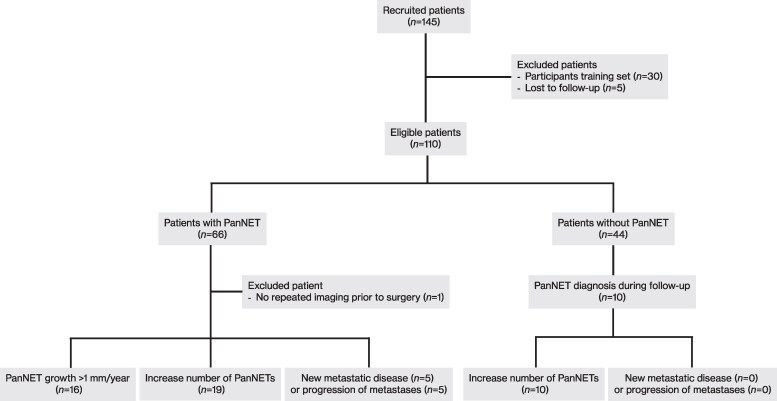
Flowchart of the study population. Abbreviation: PanNET, pancreatic neuroendocrine tumor.

Of the enrolled patients, 110 met the eligibility criteria for inclusion in the study. Five patients were excluded due to loss of follow-up within 1 year after blood sampling for the mNET assay. One patient underwent imaging only once before pancreatic surgery because of a large PanNET with locoregional lymph node metastases at baseline. Due to the unknown growth status prior to surgery, this patient was excluded from the analyses regarding the prognostic value of the mNET assay. A flowchart of the study population is shown in Fig. 1.

Baseline characteristics are summarized in Table 1. The majority were female (58%). Six (5%) patients had no identifiable germline MEN1 mutation but met the clinical criteria for MEN1. At baseline, 66 (60%) were diagnosed with a PanNET. The mean age was 46.7 years, whereas patients without a PanNET were on average 39.5 years old (*P* = .01). The median baseline mNET assay scores in patients with PanNETs was 26.7% (range 0-86.7) and 20% (range 0-100) in patients without PanNET (*P* = .74). Metastatic PanNET disease was present in 9 patients (8%) at baseline.

**Table 1.** Baseline characteristics of the study population

	n (%)		
	PanNET at baseline^*a*^ n = 66	No PanNET at baseline^*a*^ n = 44	*P*-value
**Sex**			.69
Male	29 (44)	17 (39)	
Female	37 (56)	27 (61)	
**Age, years**			
Mean (SD)	46.7 (14.2)	39.5 (14.3)	.01
**MEN1 manifestations*^b^***			
Pancreatic NET	66 (100)	0 (0)	<.001
Duodenal NET	14 (21)	3 (7)	.06
Gastric NET	5 (8)	0 (0)	.08
Lung NET	24 (36)	6 (14)	.01
Hyperparathyroidism	22 (33)	13 (30)	.84
Pituitary tumor	25 (38)	21 (48)	.33
Adrenal adenoma	26 (39)	4 (9)	<.001
**Presence of PanNET metastases**	9 (14)	0 (0)	.01
Locoregional lymph nodes	6 (9)	0 (0)	
Liver	2 (3)	0 (0)	
Liver and bone	1 (2)	0 (0)	
**History of pancreatic surgery**	13 (20)	12 (27)*^c^*	.49
Total pancreatectomy	0 (0)	4 (9)	
Whipple/PPPD + enucleation corpus/tail	1 (2)	1 (2)	
Whipple/PPPD	2 (3)	2 (5)	
Distal pancreatectomy + enucleation caput	1 (2)	0 (0)	
Distal pancreatectomy	4 (6)	4 (9)	
Enucleation caput	4 (6)	1 (2)	
Enucleation corpus/tail	1 (2)	0 (0)	
**Treatment at baseline**			
Somatostatin analogues	1 (2)	2 (5)	.56

Abbreviations: MEN1, multiple endocrine neoplasia type 1; NET, neuroendocrine tumor; PanNET, pancreatic neuroendocrine tumor; PPPD, pylorus preserving pancreaticoduodenectomy.

^*a*^Baseline is defined as the moment of blood sample collection.

^*b*^Only active manifestations are listed; those successfully treated in the past are not included in the table.

^*c*^No PanNET at baseline, after pancreatic surgery for a PanNET in the past.

### Influence of MEN1 on mNET Assay Scores

**Figure 2:**
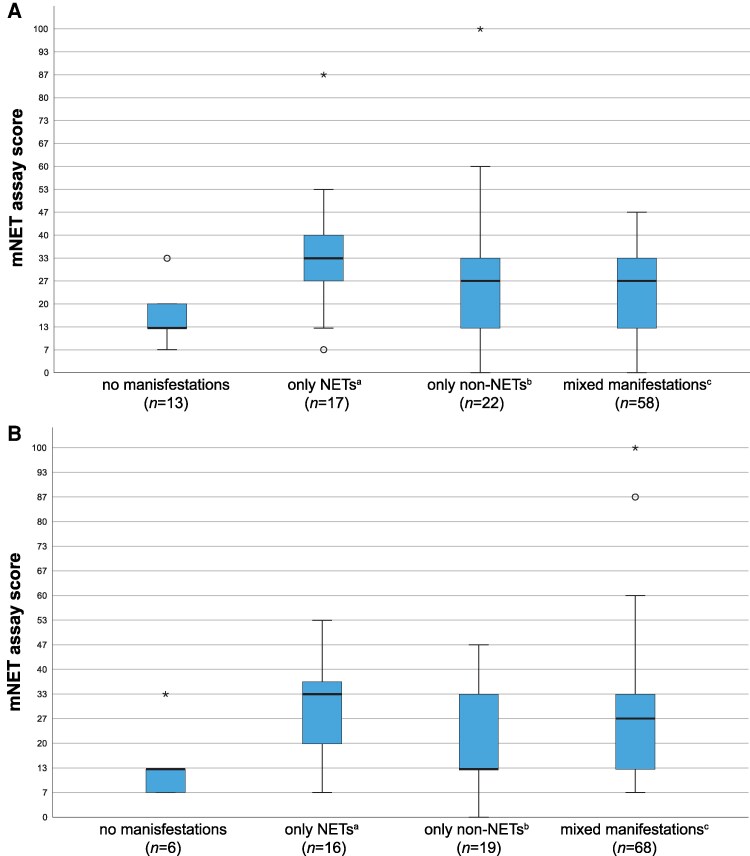
Distribution of mNET assay scores by type of MEN1 manifestations. (A) At baseline. (B) At the end of follow-up. ^a^Only NETs: pancreatic, duodenal, gastric, and/or lung NETs. ^b^Only non-NET manifestations: primary hyperparathyroidism, adrenal adenomas, and/or a pituitary tumor. ^c^Patients with both NETs and non-NET manifestations. *Outliers more than 3 times the interquartile range below the first quartile or above the third quartile. ^o^Outliers 1.5 to 3 times the interquartile range below the first quartile or above the third quartile. Abbreviations: MEN1, multiple endocrine neoplasia type 1; NET, neuroendocrine tumor.

The impact of different combinations of MEN1 manifestations on the mNET assay output was examined. Only active manifestations at the time of blood sampling were taken into account. Manifestations that had been successfully treated in the past were regarded as absent. At baseline, 13 (12%) patients had no manifestations, 22 (20%) exhibited only non-NET manifestations (eg, primary hyperparathyroidism, adrenal adenomas, and/or a pituitary tumor), 17 (15%) exclusively had NETs (pancreatic, duodenal, gastric, and/or lung NETs), and the majority (58, 53%), had both NET and non-NET manifestations, as illustrated in Fig. 2A. All 17 patients who exclusively had NETs at baseline were found to have PanNETs. Among these, 3 patients also exhibited duodenal NETs, and 5 patients had lung NETs. A Kruskal–Wallis test indicated statistically different mean ranks of the mNET assay among the groups with different combinations of MEN1 manifestations, χ^2^(3) = 8.75, *P* = .03. A post hoc analyses revealed statistically significant differences between the mNET assay scores of patients with no manifestations (mean rank = 37.6) and those with only NETs (mean rank = 70.7) (*P* = .03). There were no significant differences between the 2 groups regarding age or sex. The AUC for the mNET assay score and the presence of a NET was 0.66 [95% confidence interval (CI) .52-.81; *P* = .32], with a sensitivity of 0.77 and a specificity of 0.52. The same analysis was conducted after dividing the included patients into an additional group of those with only PanNETs (n = 10). The analysis revealed significant differences in the mNET assay scores between patients without manifestations (mean rank = 37.6) and patients with only PanNETs (mean rank = 75.8) (*P* = .04). This identifies the utility of the assay to reflect disease activity. The AUC for the mNET assay score and the presence of a PanNET was 0.52 (95% CI .41-.63; *P* = .74), with a sensitivity of 0.65 and a specificity of 0.46. There was no significant difference in mNET assay scores between patients with only NET(s) and patients with only non-NET manifestations. Additionally, no significant difference was observed in the mNET assay scores at baseline across the different mutation types.

At the end of the follow-up period, it was observed that 7 out of the 13 patients without manifestations at baseline had developed manifestations. As illustrated in Fig. 2B, there were 16 (15%) patients with only NETs, 19 (17%) patients with only non-NETs, and 68 (62%) patients with both types of manifestations. At this point, there was no longer a significant difference in mNET assay scores between the remaining 5 patients without manifestations and those with only NETs (*P* = .15). This is likely attributable to the small numbers in the latter group.

### Predictive Value for Development of PanNETs

**Figure 3:**
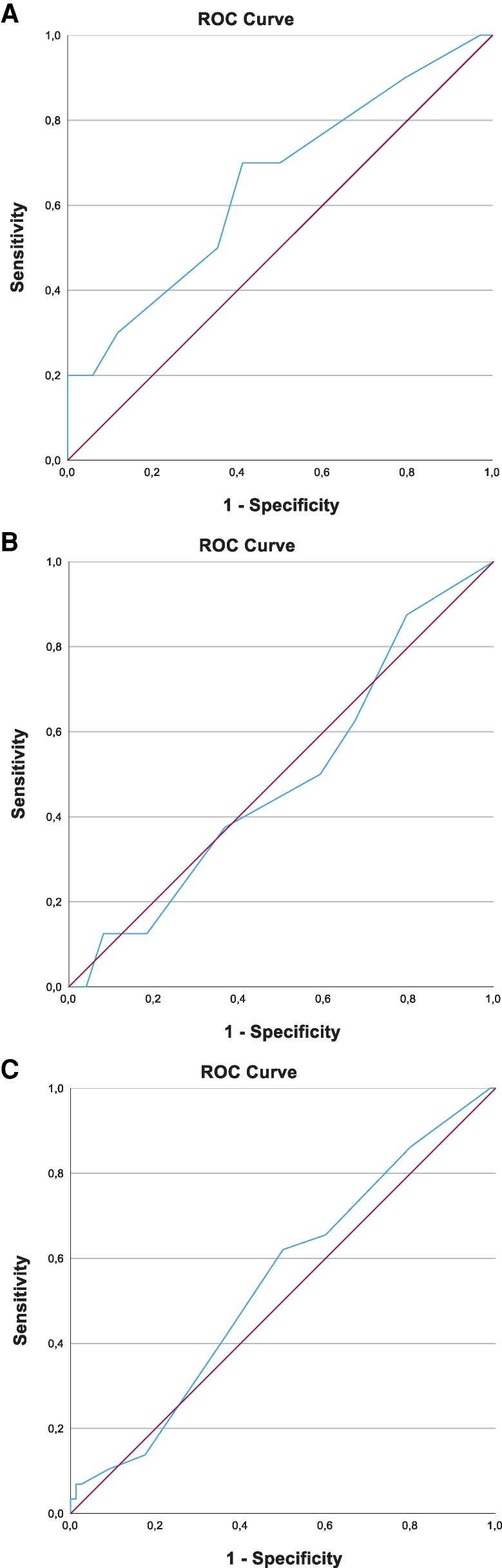

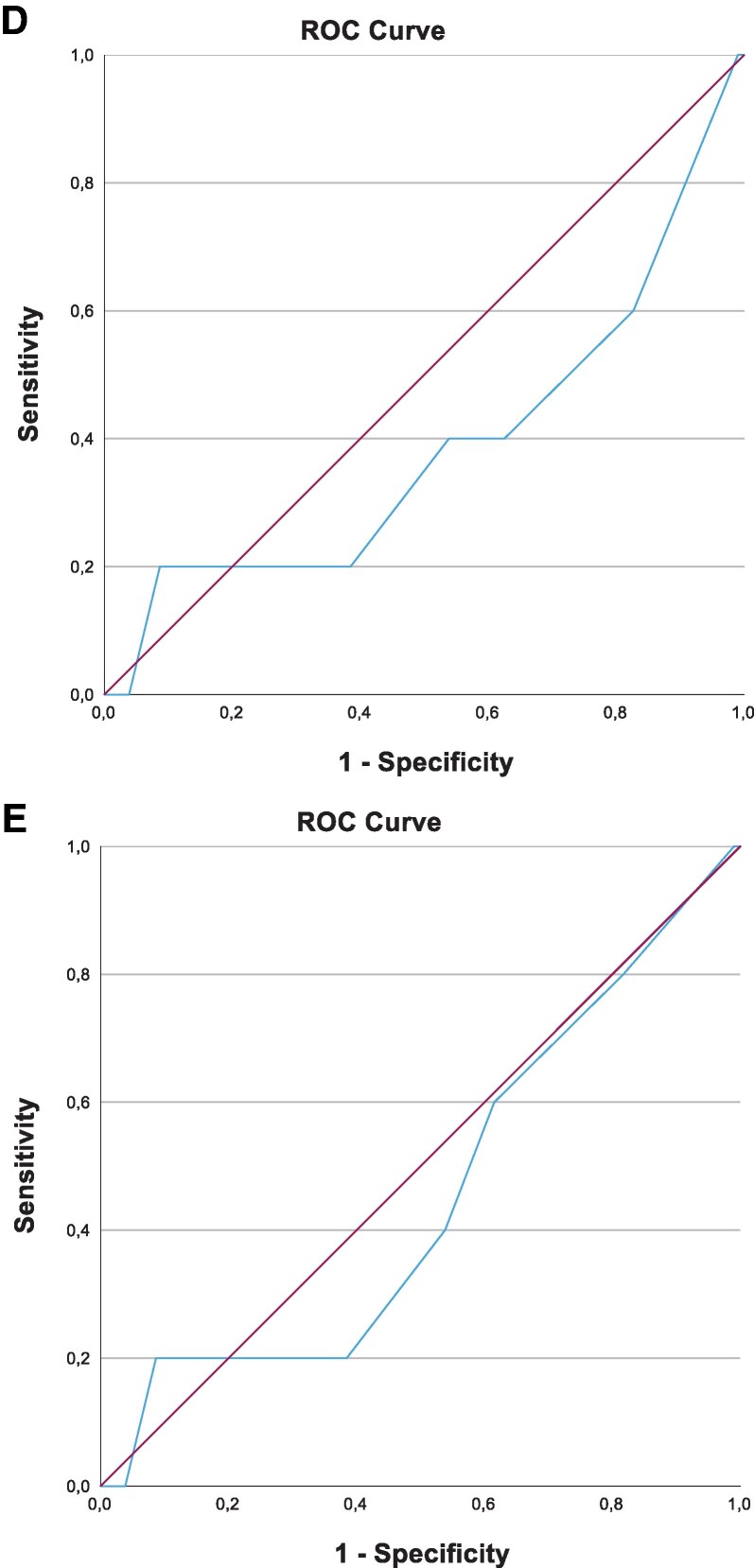
(A) The AUC for the mNET assay score to predict the development of PanNETs in MEN1 patients without PanNET at baseline was 0.65 (95% CI .45-.85; *P* = .15). (B) The AUC for predicting PanNET growth was 0.49 (95% CI .3-.65; *P* = .89). (C) The AUC for predicting an increase in the number of PanNETs was 0.54 (95% CI .42-.66; *P* = .50). (D) The AUC to predict new metastatic disease was 0.39 (95% CI .10-.68; *P* = .39). (E) The AUC to predict progression of metastases was 0.46 (95% CI .20-.71; *P* = .74). Abbreviations: AUC, area under the curve; CI, confidence interval; MEN1, multiple endocrine neoplasia type 1; mNET, multiple endocrine neoplasia type 1; PanNET, pancreatic neuroendocrine tumor.

Over the median 39-month observation period, 10 out of the 44 (23%) patients who did not have a PanNET at baseline developed a PanNET. The AUC for the mNET assay in predicting development of these PanNETs was 0.65 (95% CI .45-.85; *P* = .15). The AUC is included in Fig. 3A. The optimal cutoff point of the mNET assay score, combining sensitivity and specificity, was determined to be 20%. The metrics are displayed in Table 2.

**Table 2.** Metrics for the predictive ability of the mNET assay at 20% (as the optimal cutoff point)

	*Sensitivity (%)*	*Specificity (%)*	*PPV (%)*	*NPV (%)*
Development of PanNETs*^a^*	70.0	50.0	29.2	85.0
Growth of PanNETs*^b^*	62.5	32.7	23.3	72.7
Increase in number of PanNETs	65.5	40.0	28.4	76.2
New metastases	40.0	37.5	3.0	92.9
Progression metastases	60.0	38.5	4.5	95.2

Abbreviations: NPV negative predictive value; PanNET, pancreatic neuroendocrine tumor; PPV, positive predictive value.

^*a*^In patients without PanNET at baseline.

^*b*^In patients with PanNET at baseline.

### Predictive Value for PanNET Growth

Growth of PanNETs, defined as an increase of at least 1 mm/year, occurred in 16 patients (25%) with a PanNET at baseline. The AUC for predicting PanNET growth was calculated to be 0.49 (95% CI .33-.65; *P* = .89). Figure 3B displays the AUC. The results illustrate that, within this cohort, the mNET assay did not possess a significant predictive value for the progression of PanNETs.

### Predictive Value for an Increase in the Number of PanNETs

In 29 patients (27%), the number of PanNETs increased during the 12- to 48-month follow-up period. The AUC for the mNET assay to predict this progression in the number was 0.54 (95% CI .42–.66; *P* = .50). The optimal cutoff point of the mNET assay score was determined to be 20%. Figure 3C illustrates the AUC. The results indicate that the mNET assay does not serve as a robust predictor for the increase in the number of PanNETs in this study cohort.

### Predictive Value for New Metastatic Disease and Progression of Metastases

Five (5%) patients developed metastases, with 2 patients having locoregional lymph node metastases and 3 liver metastases. The mNET assay did not exhibit good predictive value for this indication, with an AUC of 0.39 (95% CI .10-.68; *P* = .39). Additionally, the mNET assay exhibited poor predictive value for the progression of metastases, with an AUC of 0.46 (95% CI .20-.71; *P* = .74). The AUCs are displayed in Fig. 3D and 3E.

### Overall Metrics

As a diagnostic, the mNET assay exhibited utility for detecting PanNETs in patients with MEN1 mutations. The assay also exhibited relatively good sensitivities (60-70%) for predicting growth or PanNET manifestations and had high negative predictive values (72.7-95.2%) (Table 2). This is consistent with a biomarker detecting these clinical factors. However, the metrics do not achieve levels necessary to aid with clinical decision-making.

## Discussion

In the present study, the diagnostic and prognostic utility of a recalibrated circulating gene expression assay (the mNET assay) for MEN1 was assessed in a cohort of 109 patients with a follow-up period extending up to 4 years. To our knowledge, this was the first study assessing a circulating NET mRNA assay as a biomarker for PanNETs in MEN1 patients. The strength of our study lies in the extended observation period, encompassing a maximum follow-up duration of 48 months, allowing for a more extended assessment of disease stability and progression compared to a 1-year follow-up, in a significant cohort of MEN1 patients. The current results are in line with the previous studies evaluating the original NETest in sporadic NETs that identified that the assay discriminated between those without any manifestation and patients with only PanNETs (18, 24). This indicates the validity of the outcomes of the current study. However, in MEN1, elevated scores were also observed in patients with other, non-PanNET manifestations. The involvement of (multiple) endocrine manifestations in most of our patients resulted in a limited predictive value for PanNET presence or progression, making the mNET assay as it is currently configured not clinically useful as a prognostic tool in patients with this condition.

There are several possible explanations for the limited clinical utility of the mNET assay in MEN1. The concurrent presence of multiple MEN1 manifestations in our study population may have altered the circulating RNA profile and consequently influenced the assay results. This is supported by the significant difference observed in mNET assay results between patients without any manifestations and those with only NETs. The algorithm's original training dataset comprised patients with sporadic PanNETs and did not specifically account for the simultaneous occurrence of diverse neuroendocrine neoplasms, as is characteristic in MEN1. Moreover, in MEN1, microadenomas frequently occur in the pancreas, which are too small to be detected through conventional imaging (25, 26). Although they seemingly possess minimal clinical significance, they may contribute to an elevated mNET assay score since previous studies have identified that circulating gene expression can be detected in patients with microscopic disease (including disease pathologically considered as R1) (27). This could also explain the limited positive predictive value for the development of metastatic disease, although the low number of patients experiencing new metastases may also contribute to these results. Furthermore, patients who no longer had PanNETs and locoregional lymph node metastases following pancreatic surgery (baseline sample) may harbor undetectable micrometastases. This would contribute to an increased circulating mRNA load and result in a positive NET assay score.

It is also important to acknowledge that the administration of various treatments, including somatostatin analogues (SSAs), PRRT, and surgery, may have influenced test outcomes. At baseline, 3 patients underwent SSA treatment, and an additional 4 patients initiated SSA treatment during the follow-up period. Notably, SSA treatment was primarily initiated for symptom relief in hormone-producing tumors, rather than to suppress the growth of PanNETs. Two patients with metastatic disease received PRRT, and 3 patients underwent a Whipple operation during follow-up due to progressive locoregional lymph nodes. These small numbers likely only had a minor influence on the outcomes, but it is important to acknowledge, especially since SSAs decrease circulating NET gene expression levels (28).

Another plausible explanation for the results involves the different patterns of epigenetic modifications between sporadic PanNETs and those in MEN1 patients, leading to differences in circulating mRNA profiles. The MEN1 gene encodes menin, a multifunctional protein that plays a key pathophysiological role in epigenetic regulation and gene transcription. Menin influences the expression of genes involved in cell proliferation, contributing significantly to tumorigenesis (29-31). In the MEN1 syndrome, there is a high rate of hypermethylation patterns that are different from those observed in sporadic PanNETs and that contribute to alterations in gene expression and variations in phenotype (32-35). The dissimilar patterns of DNA methylation between MEN1 patients and individuals with sporadic PanNETs could contribute to the differences in prognostic scores between the mNET assay and the NETest. Moreover, the high rate of DNA methylation may offer an explanation for the weak genotype-phenotype correlation in MEN1. However, it is precisely within this group of patients where there is a significant demand for tests capable of predicting the course of the disease, given the substantial burden of regular follow-up scans. Further research and validation studies are imperative to elucidate the epigenetic-phenotype correlations, thereby potentially improving circulating gene expression tests like the mNET assay. Alternatively, identifying novel mRNA targets for MEN1 prognostic assays may also be a viable option.

Lastly, in contrast to the majority of studies assessing the original NETest, in this study blood was collected in PAXgene tubes. We tested the correlation between EDTA-based gene expression and PAXgene gene expresion intraindividually in a small subgroup, and this was low (data not published). This identifies that the original NETest EDTA-based algoritms would not work in PAXgene collected samples. This left us to recalibrate the algorithms for the PAXgene samples. By using both healthy controls (n = 23), sporadic gastroenteropancreatic NETs (n = 5), and a training set of MEN1 patients (n = 30), we attempted to recalibrate the assay as reliably as possible. However, the training set is relatively small, which could have influenced the validation set results.

In conclusion, this study revealed the limited predictive efficacy of the mNET assay as a prognostic biomarker in individuals with MEN1. In contrast, the test has shown promise for detecting PanNETs in MEN1. We anticipate that optimization of the machine learning algorithms would improve the predictive value for the development and progression of PanNET metastases. Further research is imperative for the development of reliable biomarkers in MEN1 to enable early detection of disease progression, optimizing personalized management and facilitating timely interventions for PanNETs in MEN1.

## Data Availability

Some or all datasets generated during and/or analysed during the current study are not publicly available but are available from the corresponding author on reasonable request.
